# Pyrvinium doubles against WNT-driven cancer

**DOI:** 10.1016/j.jbc.2022.102479

**Published:** 2022-09-09

**Authors:** Jiaming Fan, Russell R. Reid, Tong-Chuan He

**Affiliations:** 1 Ministry of Education Key Laboratory of Diagnostic Medicine, and Department of Clinical Biochemistry, The School of Laboratory Medicine, Chongqing Medical University, Chongqing, China; 2 Molecular Oncology Laboratory, Department of Orthopaedic Surgery and Rehabilitation Medicine, The University of Chicago Medical Center, Chicago, Illinois, USA; 3 Laboratory of Craniofacial Suture Biology and Development, Department of Surgery Section of Plastic Surgery, The University of Chicago Medical Center, Chicago, Illinois, USA

**Keywords:** WNT/β-catenin signaling, casein kinase 1α, E3-ubiquitin ligase component Cereblon, WNT-driven cancer, anti-WNT agents, repurposed drugs, pyrvinium, CK1α activator

## Abstract

The WNT–β-catenin signaling pathway has a major role in regulating cell proliferation and differentiation. Aberrant activation of the pathway contributes to various human cancer types. Because casein kinase CK1α-initiated phosphorylation of β-catenin is a key first step to restrain WNT signaling, effective restoration of CK1α activity represents an innovative strategy to combat WNT-driven cancer. A recent study in JBC reveals the anthelmintic pyrvinium directly binds to CK1α as an activator and also stabilizes CK1α protein, doubling against WNT-driven cancer activity.

Since the first *Wnt* gene discovery about 40 years ago, evolutionarily conserved Wnt signaling has attracted tremendous attention due to its essential roles in regulating embryonic development and governing stem cell proliferation and differentiation (1). The signaling pathway is activated by a family of 19 lipoglycoproteins called WNTs in humans, whose production, secretion, and diffusion through tissues are tightly regulated. Depending on the ligand-receptor combination, cellular context, and downstream events regulated, Wnt signaling is transmitted through two overlapping but distinct pathways: the β-catenin–independent pathway (*i.e.*, noncanonical pathway) and the β-catenin–dependent pathway (canonical pathway). While less understood, the noncanonical pathway is involved in cytoskeleton rearrangement, cell polarity, and migration (also known as WNT/planar cell polarity or WNT/PCP branch) or in promoting proliferation and antagonizing the canonical pathway through the WNT/Ca^2+^ branch. On the other hand, strongly associated with human diseases and cancer, the canonical pathway has been the focus of most WNT-related studies. In this pathway, WNT binds to one of 10 Frizzled (FZD) receptors and the coreceptors LDL-receptor–related proteins 5 and 6 (LRP5 and LRP6), transduces the signal through the FZD-associated scaffolding protein Dishevelled (DVL), and recruits binding protein AXIN to the cytoplasm membrane, leading to the release of β-catenin from the dissembled destruction complex containing AXIN, adenomatous polyposis coli (APC), glycogen synthase kinase 3β, casein kinase 1α (CK1α), the E3-ubiquitin ligase β-TrCP, and protein phosphatase 2A (2). β-Catenin is thus stabilized and accumulates in the cytoplasm, which then translocates to the nucleus to bind with the transcription factors T-cell factor/Lymphoid enhancer factor (TCF/LEF) and regulates downstream genes such as *c-Myc* and *cyclin D1* by displacing TCF/LEF-bound corepressors and/or recruiting coactivators (Fig. 1) (2).

**Figure 1:**
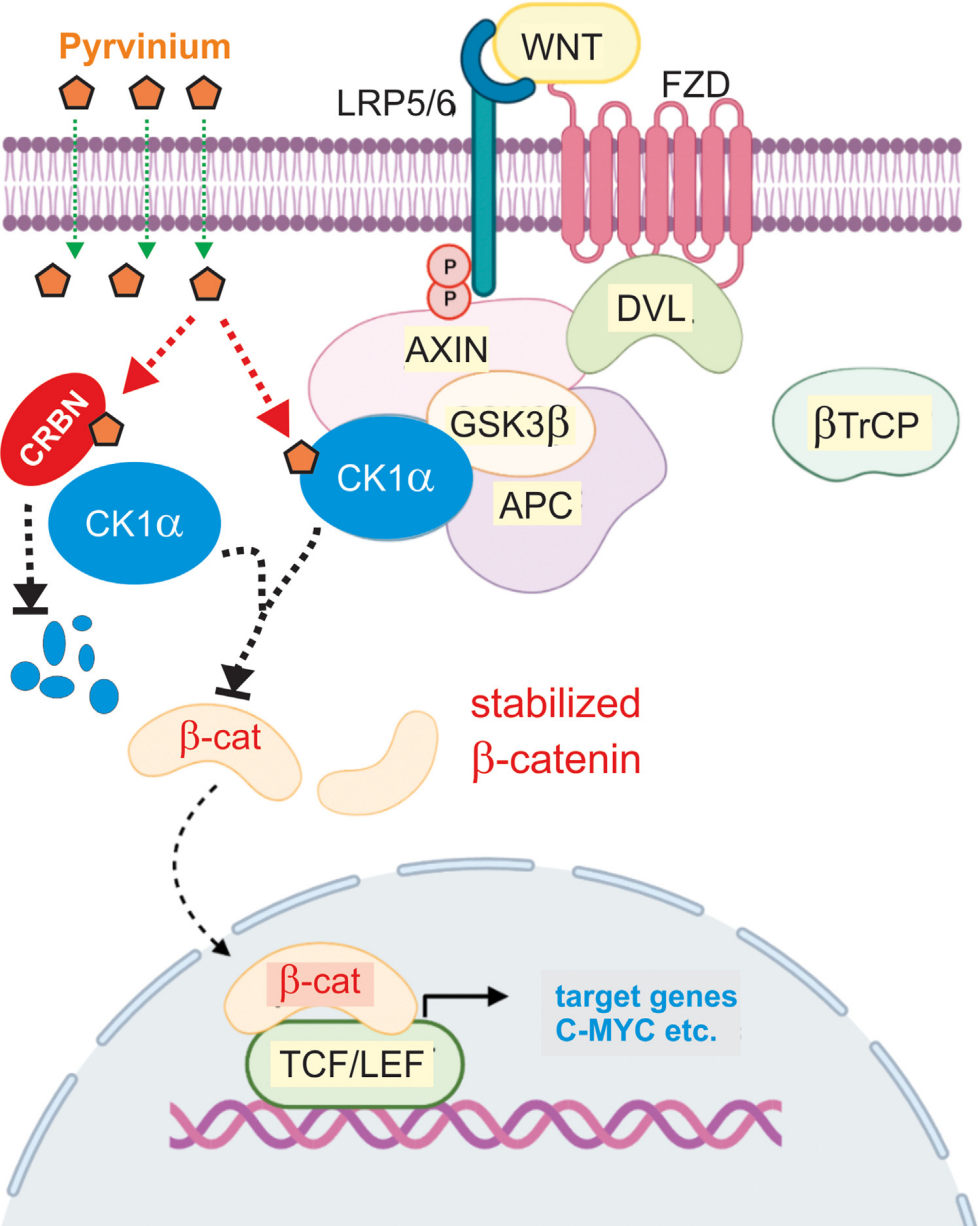
**Dual mechanism model of anthelmintic pyrvinium in CK1α-controlled WNT signaling.** Canonical WNT binds to its receptors FZD and LRP5/6 and triggers the disassembly of destruction complex containing CK1α, leading to stabilization of β-catenin protein. WNT signaling also induces CK1α degradation through Cereblon (CRBN). Anthelmintic pyrvinium not only directly binds to CK1α as an activator but also stabilizes CK1α protein thus to exert its potent anti-WNT and anticancer activity.

The essence of canonical WNT signaling activation is the stabilization of β-catenin protein. Effective elimination of free cytoplasmic β-catenin is the key to keeping the canonical pathway in check under physiological conditions. The “priming” step of β-catenin degradation involves the CK1α-initiated phosphorylation of β-catenin at its Ser45 residue, which enables glycogen synthase kinase 3β to phosphorylate the Ser33, Ser37, and Thr41 residues, creating the binding site for the adapter protein β-TrCP that complexes with other proteins to promote β-catenin ubiquitination and subsequent proteasomal destruction. Interestingly, as a critical WNT negative regulator, CK1α stability is negatively regulated by WNT through E3-ubiquitin ligase component Cereblon (CRBN)-initiated degradation (3). This regulatory loop squarely establishes a prominent position of CK1α in the tight control of canonical WNT signaling, suggesting that restoration of CK1α kinase activity and/or stabilization of CK1α protein may be exploited if canonical WNT signaling is aberrantly activated.

Perhaps, upward of half of all human solid tumors and leukemias harbor aberrant constitutive activation of canonical WNT signaling through genetic and/or epigenetic alterations of the key players in the pathway, such as loss-of-function mutations of *APC* or *AXIN* or gain-of-function/oncogenic mutations of *β-catenin* (4, 5). Thus, for the past decades, significant efforts have been devoted to devising WNT-targeting strategies as potential innovative anticancer therapeutics, such as the use of the naturally occurring secreted FZD-related proteins (serving as FZD decoy receptors), anti-FZD antibodies, Porcupine (PORCN) inhibitors (preventing the posttranslational palmitoylation of WNT ligands for efficient secretion), tyrosine kinase–like orphan receptor (ROR) inhibitors (blocking the WNT/ROR noncanonical pathway), and Tankyrase inhibitors (blocking the degradation of AXIN), to name a few (5). While some of these have shown promising anticancer activities in preclinical studies, there is a long road ahead for their eventual clinical use.

Repurposing current FDA-approved drugs is a time-saving and cost-effective alternative to cancer drug discovery. One such FDA-approved drug, pyrvinium, was identified as a potent inhibitor of WNT signaling through a biological function-based screen for compounds that could stabilize AXIN and promote β-catenin turnover in *Xenopus laevis* eggs (6). Mechanistically, pyrvinium was shown to directly bind to CK1α and allosterically activate its kinase activity, leading to enhanced β-catenin degradation and hence inhibition of Wnt signaling (Fig. 1). Pyrvinium (Viprynium) is a quinoline-derived cyanine dye with anthelmintic properties effective against pinworms and was approved by the FDA in the 1950s (7). Pyrvinium’s anthelmintic activity is attributed to its interference with glucose uptake and/or inhibition of mitochondrial respiration complex 1 and the unfolded protein response in pinworms (7). Since its initial discovery as a CK1α activator, pyrvinium has been shown to inhibit tumor growth in various types of cancer (8).

More recently, Shen *et al.* (9) revealed a new mechanism through which pyrvinium attenuates Wnt-induced CK1α degradation *via* interaction with the E3-ubiquitin ligase component Cereblon. Specifically, through a series of biochemical and cell biology assays, pyrvinium was shown to disrupt the interaction between Cereblon and CK1α at the immunomodulatory drug– binding pocket within the Cereblon–CK1α complex (Fig. 1), which was independent of the direct binding of pyrvinium to CK1α as an agonist as discussed above (9). These interesting findings reveal a novel dual mechanism through which pyrvinium can effectively inhibit canonical WNT signaling by attenuating the CRBN-mediated degradation of CK1α while also directly activating CK1α kinase activity, further justifying the repurposed use of pyrvinium as an anticancer agent to treat WNT-driven cancers and/or any cancer harboring aberrant activation of canonical WNT signaling. It is noteworthy that a targeted restoration of CK1α activity should provide an enhanced therapeutic index for CK1α activators like pyrvinium, as it has been reported that CK1α abundance is decreased in WNT-driven tumors relative to normal tissue (10).

While the evidence surrounding pyrvinium’s activation of CK1α and subsequent inhibition of WNT signaling is convincing, it is conceivable that, like many other small molecule drugs, pyrvinium may exert its anticancer activity by targeting other cellular processes in addition to WNT. Pyrvinium indeed has been shown to target mitochondria under nutrient-depleted conditions, impair JAK/STAT signaling, downregulate AKT/PKB signaling, or target autophagy addiction as a part of its anticancer mechanisms, which indicates pyrvinium might be a home run. However, a major hurdle for pyrvinium’s clinical anticancer use is its low bioavailability, which may demand innovative delivery strategies. Nonetheless, pyrvinium presents an exciting prospect of being one of the first potent WNT-targeting agents for cancer treatment.

## Conflict of interest

The author declares that they have no conflicts of interest with the contents of this article.
